# Trends in psychotropic use among older adults with dementia in Korean long-term care hospitals across the COVID-19 pandemic: a national longitudinal study

**DOI:** 10.3389/fpubh.2026.1776066

**Published:** 2026-06-15

**Authors:** Eunjin Kim, Jung Min Yoon, Alison M. Trinkoff

**Affiliations:** 1 College of Nursing, Ewha Womans University, Seoul, Republic of Korea; 2 School of Nursing, University of Maryland, Baltimore, MD, United States

**Keywords:** COVID-19 pandemic, dementia, long-term care hospital, older adult, psychotropic drugs

## Abstract

**Background:**

Disruptions to routine care and non-pharmacological interventions during the COVID-19 pandemic placed significant stress on dementia care in South Korea’s long-term care (LTC) hospitals, which operate under a medicalized, insurance-integrated structure with high reliance on pharmacologic symptom management. However, limited research has examined whether psychotropic use increased in Korean LTC hospitals during the pandemic and whether changes persisted after the early pandemic period among older adults with dementia.

**Methods:**

A national longitudinal study was conducted using Korean National Health Insurance Service claims data for 385,602 older adults (≥ 65 years) with dementia hospitalized in LTC hospitals from 2019, Quarter 4 to 2021, Quarter 4. Quarterly psychotropic use was assessed using prescription days and defined daily doses (DDDs), each standardized per 100 inpatient-days. Linear mixed models with LTC-hospital random intercepts were used to estimate quarterly trends, adjusting for facility and patient-level factors.

**Results:**

Compared with the 2019Q4 baseline, adjusted prescription days and DDDs increased for all psychotropic classes (all *p* < 0.0001). Antipsychotic medications showed the largest increases, peaking in 2021Q1 [2.67 days (95% CI 2.26–3.12) and 0.57 DDDs (95% CI 0.48–0.68)] and remaining above baseline in 2021Q4 [0.27 days (95% CI 0.06–0.50) and 0.09 DDDs (95% CI 0.03–0.16)], although diminishing in the later quarters. Anxiolytics and antidepressants also increased, with larger early-pandemic rises for anxiolytics [e.g., 2020Q1: 0.17 days (95% CI 0.12–0.22) and 0.10 DDDs (95% CI 0.07–0.14)] and steady increases for antidepressants through 2021Q4 [0.14 days (95% CI 0.10–0.19) and 0.09 DDDs (95% CI 0.07–0.12)].

**Conclusion:**

Psychotropic use remained elevated above pre-pandemic baseline levels through 2021, although increases attenuated substantially in the later quarters, particularly for antipsychotics. These findings suggest increased reliance on pharmacologic symptom management when routine psychosocial dementia care was constrained. Strengthening crisis-resilient, person-centered dementia care in LTC hospitals, including continuity of non-pharmacological interventions, staffing supports, and psychotropic stewardship and monitoring beyond antipsychotics, may reduce psychotropic reliance during public health emergencies.

## Introduction

Dementia care in institutional long-term care (LTC) settings depends on system capacity to deliver person-centered, non-pharmacological approaches for behavioral and psychological symptoms of dementia (BPSD). Although non-pharmacological interventions are recommended as first-line management for BPSD (1, 2), many LTC settings often rely on psychotropic drugs, despite concerns about adverse effects and limited efficacy for certain symptoms (3, 4). These concerns have driven stewardship initiatives to reduce inappropriate antipsychotic use and strengthen the quality of dementia care across countries, including the U. S. CMS National Partnership to Improve Dementia Care in Nursing Homes and Canada’s national monitoring program on antipsychotic prescribing (5, 6).

In South Korea (hereafter Korea), the prevalence of dementia is projected to rise from 10% in 2019 (approximately 830,000 people) to 16% by 2050 (around 3.02 million people), particularly among adults aged ≥ 65 years (7). According to a national health-economic analysis, Korea’s total economic burden of dementia was estimated at USD 4.22 billion in 2019, representing a 1.5-fold increase since 2015, with a per-capita cost of approximately USD 6,957 (8). To address the growing burden of chronic and cognitive conditions, LTC hospitals were established as medicalized, insurance-integrated institutions to provide medical treatment and nursing care for older adults with chronic illnesses, disabilities, and/or cognitive impairment (9). People diagnosed with dementia account for the largest proportion of the care population across LTC settings (10, 11), and caring for BPSD remains a major challenge, given that over 80% of residents in LTC settings experience one or more BPSD (12, 13). Effective management of BPSD is a central component of dementia care, requiring LTC hospitals and staff to adapt to the increasing clinical complexity and care needs of residents with dementia.

To address concerns about inappropriate psychotropic use, Korea introduced a new LTC hospital quality indicator related to psychotropic use, effective November 1, 2019 (14). Shortly thereafter, the first COVID-19 case in Korea was announced in January 2020, followed by the first outbreak in an LTC hospital in February 2020 (15, 16). Outbreaks in LTC hospitals comprised 13% of all recorded COVID-19 cases in Korea and over one-third (35.1%) of total COVID-19 deaths (16, 17). The pandemic substantially altered LTC hospital operations, characterized by staffing constraints, restricted visitation, and social distancing measures that likely limited the delivery of psychosocial care and strained routine quality-monitoring processes. The pandemic and related LTC changes may have further challenged the delivery of quality care for residents with dementia and exacerbated their psychological distress and overall well-being (18). This context provides a natural stress test of whether a psychotropic stewardship-related quality initiative was maintained during the crisis.

While previous studies in North America and Europe reported increases in psychotropic use among residents with dementia in LTC settings during the pandemic (19–21), little is known about psychotropic prescribing patterns in Korean LTC hospitals. A recent nationwide interrupted time series analysis using Korean National Health Insurance Service (NHIS) claims data reported increased antipsychotic prescribing among older adults with dementia during the pandemic. However, the study included patients across all care settings except those hospitalized in LTC hospitals throughout the study period, and the scope was restricted to antipsychotics alone (22). According to a systematic review and meta-analysis on psychotropic use for BPSD during the pandemic, although antipsychotic use predominantly increased, some primary studies also reported increases in anxiolytic and antidepressant use (23). Primary investigation of psychotropic classes beyond antipsychotics among older adults with dementia in Korean LTC hospitals during the pandemic is warranted.

Thus, this national longitudinal study using Korean NHIS claims data aimed to examine quarterly changes in psychotropic use, including antipsychotics, anxiolytics, and antidepressants, among older adults aged ≥ 65 years with dementia across Korean LTC hospitals in relation to the pandemic. Linear mixed models with LTC-hospital random intercepts were applied to capture quarter-specific patterns of change, adjusting for facility and patient-level factors. We hypothesized that prescription days and doses for each psychotropic class would increase during the pandemic period compared with the pre-pandemic baseline, and remain elevated after the early pandemic period.

## Materials and methods

### Study design and data source

A longitudinal design was employed for the study. This study utilized de-identified, population-based data extracted from the NHIS claims databases in Korea. The NHIS operates as a compulsory, single-payer health insurance system that covers approximately 97% of the Korean population (24). The NHIS claims data include detailed information on subject demographics, diagnostic codes, treatment records (except non-reimbursable services), and healthcare institutional characteristics. Upon the author’s request, the NHIS provided customized data for older adults diagnosed with dementia who were hospitalized in LTC hospitals during the study period (NHIS data provision approval: REQ202303151-022).

### Study period

The observation period spanned from the fourth quarter of 2019 (2019Q4; October 1–December 31, 2019) to the fourth quarter of 2021 (2021Q4), covering both the pre-pandemic and pandemic periods. We designated 2019Q4 as the reference period, representing the pre-pandemic baseline. The pandemic began in 2020Q1, with the first confirmed case on January 20, 2020 (15). Visitor restrictions in LTC hospitals began on February 17, 2020, with a complete visitor ban on March 20, 2020 (25).

### Study population

People with dementia were identified using diagnostic codes from the seventh and eighth editions of the Korean Standard Classification of Diseases (KCD-7 and KCD-8; KCD-7 until December 31, 2020; KCD-8 from January 1, 2021), as defined in the 2021 National Dementia Quality Assessment Implementation Plan by the Health Insurance Review and Assessment Service (HIRA) (26). The diagnostic codes included: dementia in Alzheimer’s disease (F00), vascular dementia (F01), dementia in other diseases classified elsewhere (F02), unspecified dementia (F03), delirium superimposed on dementia (F051), Alzheimer’s disease (G30), behavioral variant frontotemporal dementia (G3100), semantic variant primary progressive aphasia (G3101), nonfluent primary progressive aphasia (G3102), logopenic primary progressive aphasia (G3103), primary progressive aphasia not elsewhere classified (G3104), and dementia with Lewy bodies (G3182).

**Figure 1:**
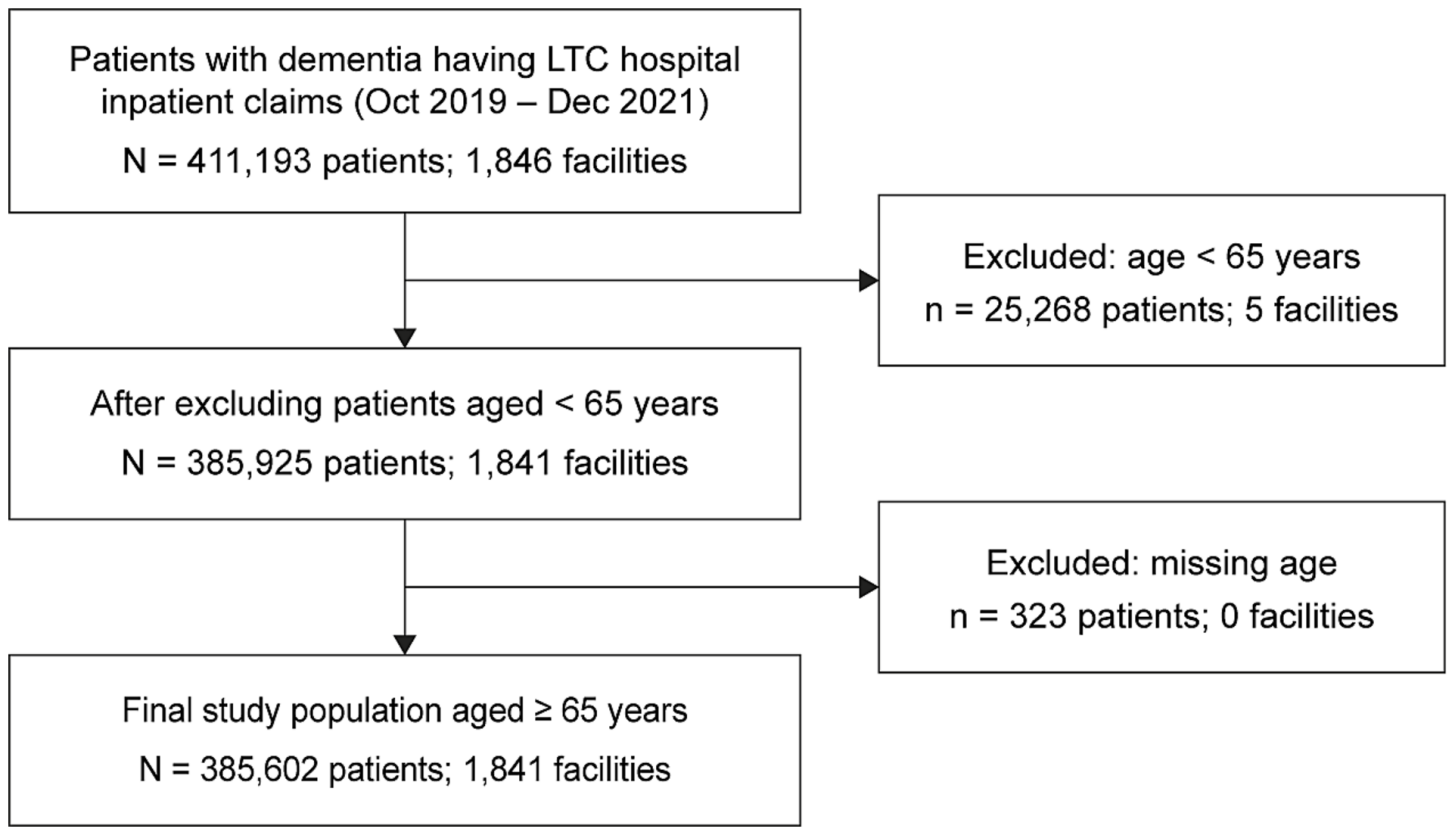
Flow diagram of study population selection.

Using the NHIS claims database, which captures all inpatient claims submitted by LTC hospitals in Korea, 411,193 patients diagnosed with dementia who had inpatient claims from 1,846 LTC hospitals were identified between October 1, 2019, and December 31, 2021. We sequentially excluded 25,268 patients aged < 65 years (along with 5 facilities that had no remaining eligible patients) and 323 patients with missing age data. This yielded a final study population of 385,602 hospitalized patients with dementia aged ≥ 65 years from 1,841 LTC hospitals across all study quarters (Figure 1).

### Measures

#### Study outcomes: psychotropic use

The primary outcome variable was psychotropic use, assessed quarterly using two metrics, each standardized per 100 inpatient-days to account for differences in LTC hospital census: *prescription days*, defined as the total number of days with a prescription for psychotropics within a quarter (regardless of dose or frequency), and *prescription doses*, defined as total quarterly doses expressed as defined daily doses (DDDs). Both measures were calculated at the patient level and summarized within each LTC hospital.

The DDD refers to the assumed average daily maintenance dose of a drug used for its primary indication in adults (27). For each drug, prescription doses were converted into DDDs using definitions from the WHO Collaborating Centre for Drug Statistics Methodology (28). However, two drugs (etizolam and blonanserin) lacked WHO-defined DDD values; thus, their prescription doses were treated as missing in DDD-based analyses.

Psychotropics were identified among reimbursed “neuropsychiatric drugs” (Ministry of Food and Drug Safety classification code 117) (29) and grouped into three classes using the WHO Anatomical Therapeutic Chemical (ATC) Classification System: antipsychotics, anxiolytics, and antidepressants (28). Additionally, prescription days and DDDs were examined for frequently prescribed drugs within each class:

- *Antipsychotics*: aripiprazole, clozapine, haloperidol, olanzapine, quetiapine, risperidone
- *Anxiolytics*: alprazolam, diazepam, lorazepam
- *Antidepressants*: escitalopram, mirtazapine, sertraline, trazodone

#### Covariates

Covariates included facility and patient-level factors that were potentially associated with psychotropic use. Facility-level factors included region (metropolitan, medium/small cities, or medically underserved areas), bed capacity, bed-to-registered nurse (RN) ratio, and bed-to-nursing assistant (NA) ratio. Medically underserved areas were defined as government-designated regions under Article 7 (4) of the Enforcement Rule of the Income Tax Act (30), characterized by geographic remoteness (e.g., islands, mountainous regions) and/or limited access to healthcare.

Patient-level factors included gender, age, the number of chronic diseases per quarter (range: 0–13), presence of psychiatric diseases (yes/no), and presence of neurological diseases (yes/no), with dementia excluded from all disease-related variables. Chronic, psychiatric, and neurological diseases were defined using diagnostic codes from KCD-7 and KCD-8 according to the Standardized Guide to Disease and Medical Practice Statistical Calculations by the HIRA (31). COVID-19 was also included among the chronic diseases (32). Detailed KCD codes are provided in Supplementary Table S1.

### Statistical analysis

The quarterly characteristics of LTC hospitals and older adults with dementia were summarized using frequencies and percentages for categorical variables and means and standard deviations for continuous variables. For variables with absolute skewness or kurtosis values > 2, medians and interquartile ranges (IQRs) were also reported.

Linear mixed models with random intercepts for LTC hospitals were employed to examine quarterly changes in psychotropic prescription days and doses (DDDs) among older adults with dementia from 2019Q4 to 2021Q4. Given that the intraclass correlation coefficients (ICCs) among patients within each LTC hospital exceeded 1% in the null models (33), LTC hospitals were included as random effects to account for intra-facility correlations. Models included quarter indicators and were adjusted for the facility and patient-level covariates described above.

Because diagnostic assessments of residuals indicated non-normality, prescription days and DDDs were natural log-transformed after adding a constant of 0.1 to accommodate zero values. Least squares means (LS-means) for each quarter were estimated from the fitted linear mixed models and back-transformed to the original scale. Mean differences were then calculated by comparing each quarter’s back-transformed LS-means with those of the reference quarter (2019Q4), representing absolute changes in days or DDDs per 100 inpatient-days. To address multiple pairwise comparisons across nine quarters, a Bonferroni correction was applied with an adjusted significance level of *α* = 0.0013 (0.05/36). All statistical analyses were performed using SAS software, version 9.4 (SAS Institute Inc., Cary, NC, United States).

### Ethical considerations

This study was exempted from review by the Institutional Review Board of the principal investigator’s institution (****-202311–0005-01). Thereafter, the authors obtained approval from the NHIS Committee for the Review of National Health Information Data Provision for data access (NHIS-2024-1-371).

## Results

### Quarterly characteristics of LTC hospitals and older adults with dementia

Across quarters, 1,526–1,639 LTC hospitals were included, reflecting LTC hospitals with at least one eligible patient in that quarter (Table 1). Approximately 55% were located in medium or small cities, and hospitals with 100–199 beds were most common (52–57%). Median bed-to-RN ratio decreased from 10.91 (IQR: 8.10, 13.82) in 2019Q4 to 10.35 (IQR: 7.50, 13.17) in 2021Q4; the median bed-to-NA ratio declined from 8.29 (IQR: 6.89, 11.43) to 8.10 (IQR: 6.81, 10.42).

**Table 1:** Quarterly characteristics of LTC hospitals and older adults with dementia.

Characteristics	2019Q4 (Oct–Dec)	2020Q1 (Jan–Mar)	2020Q2 (Apr–Jun)	2020Q3 (Jul–Sep)	2020Q4 (Oct–Dec)	2021Q1 (Jan–Mar)	2021Q2 (Apr–Jun)	2021Q3 (Jul–Sep)	2021Q4 (Oct–Dec)
LTC hospitals, *N*	1,532	1,532	1,526	1,535	1,526	1,543	1,615	1,633	1,639
Region, %									
Metropolitan	39.2	39.4	39.2	39.4	39.3	39.3	39.5	39.5	40.0
Medium/small	55.6	55.6	55.8	55.7	55.7	55.9	55.2	55.2	54.7
Medically underserved	5.2	5.0	5.1	4.9	5.0	4.8	5.3	5.3	5.3
Bed capacity, %									
< 50	5.9	5.5	5.5	5.5	5.6	6.0	8.4	8.5	8.7
50–99	12.3	11.7	11.9	11.8	11.7	10.9	11.9	12.1	12.1
100–199	57.2	55.9	55.9	55.7	56.0	55.3	53.1	52.6	51.9
≥ 200	23.6	26.3	26.1	26.2	26.0	27.2	25.8	25.5	25.4
Bed-to-RN ratio									
M ± SD	11.95 ± 7.92	11.34 ± 6.26	11.40 ± 6.37	11.38 ± 6.39	11.41 ± 6.40	11.10 ± 6.36	10.85 ± 6.34	10.80 ± 6.33	10.78 ± 6.37
Median (IQR)	10.91 (8.10, 13.82)	10.64 (7.96, 13.54)	10.67 (7.96, 13.55)	10.64 (7.96, 13.42)	10.65 (8.00, 13.54)	10.53 (7.93, 13.33)	10.42 (7.56, 13.27)	10.36 (7.56, 13.22)	10.35 (7.50, 13.17)
Bed-to-NA ratio									
M ± SD	10.91 ± 11.72	10.18 ± 10.52	10.20 ± 10.65	10.18 ± 10.47	10.15 ± 10.49	9.34 ± 6.24	9.10 ± 4.83	9.13 ± 4.92	9.17 ± 5.29
Median (IQR)	8.29 (6.89, 11.43)	8.05 (6.83, 10.82)	8.07 (6.83, 10.88)	8.13 (6.86, 11.00)	8.09 (6.86, 10.88)	8.18 (6.93, 10.44)	8.12 (6.83, 10.38)	8.13 (6.83, 10.42)	8.10 (6.81, 10.42)
Older adults with dementia, *N*	193,521	190,928	185,097	184,268	179,665	177,986	180,076	180,751	178,732
Female, %	72.4	72.1	72.2	71.9	72.0	71.5	71.0	70.8	70.9
Age, M ± SD	82.86 ± 7.20	83.52 ± 7.37	83.37 ± 7.35	83.20 ± 7.33	83.05 ± 7.32	83.67 ± 7.50	83.43 ± 7.53	83.27 ± 7.51	83.16 ± 7.48
No. of chronic diseases, M ± SD^†^	3.06 ± 1.57	3.08 ± 1.57	3.08 ± 1.57	3.09 ± 1.57	3.10 ± 1.57	3.12 ± 1.57	3.13 ± 1.58	3.13 ± 1.58	3.17 ± 1.59
Presence of psychiatric diseases, %^†^	35.1	36.0	36.0	36.5	36.9	37.7	38.8	38.6	39.2
Presence of neurological diseases, %^†^	47.6	47.9	47.7	47.7	47.9	48.1	47.9	47.6	47.6

^†^Excluding dementia.

IQR, interquartile range; LTC, long-term care; M, mean; NA, nursing assistant; RN, registered nurse; SD, standard deviation.

The quarterly number of older adults with dementia hospitalized in LTC hospitals ranged from 177,986 to 193,521. Females consistently comprised approximately 70% of the population, with a mean age of about 83 years. Other than dementia, patients had an average of three chronic diseases. Between 2019Q4 and 2021Q4, the prevalence of psychiatric diseases increased from 35.1 to 39.2%, representing an 11.7% increase, while neurological diseases remained stable at approximately 48%.

### Changes in psychotropic use for older adults with dementia in LTC hospitals

#### Prescription days

**Figure 2:**
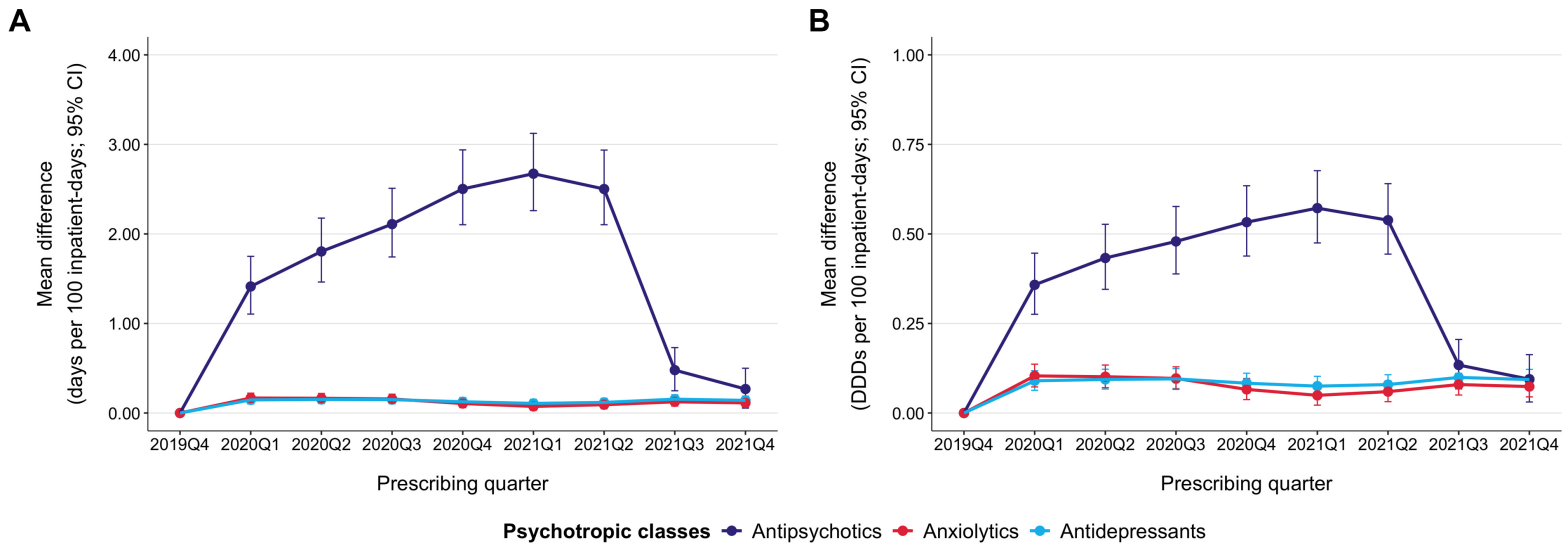
Adjusted changes in prescription days and doses by psychotropic class compared with the 2019Q4 baseline (per 100 inpatient-days). **(A)** Prescription days. **(B)** Defined daily doses (DDDs). CI = confidence interval; DDDs = defined daily doses. All mean differences were statistically significant compared to 2019Q4 (*p* < 0.0001; Bonferroni-corrected *α* = 0.0013). Circles represent mean differences; error bars represent 95% CIs.

After adjusting for facility and patient-level factors, prescription days increased for all psychotropic classes in every quarter compared with the pre-pandemic baseline (Table 2; Figure 2A; all *p* < 0.0001). The magnitude of increase was greatest for antipsychotics, followed by anxiolytics and antidepressants. Antipsychotic increases peaked in early 2021 and remained above baseline through 2021Q4, although diminishing in later quarters. Anxiolytic prescription days increased across all quarters, with the greatest rise in early 2020, while antidepressant days rose steadily throughout the study period. Drug-specific results are provided in Supplementary Table S2 and Supplementary Figures S1A–S3A.

**Table 2:** Adjusted linear mixed model estimates of changes in prescription days by psychotropic class compared with the 2019Q4 baseline (per 100 inpatient-days).

Prescribing quarter	Antipsychotics	Anxiolytics	Antidepressants
	Mean difference (95% CI) ^a^	Mean difference (95% CI) ^a^	Mean difference (95% CI) ^a^
2020Q1	1.41 (1.11, 1.75)	0.17 (0.12, 0.22)	0.15 (0.10, 0.20)
2020Q2	1.80 (1.46, 2.18)	0.17 (0.12, 0.22)	0.15 (0.11, 0.20)
2020Q3	2.11 (1.74, 2.51)	0.16 (0.11, 0.21)	0.15 (0.10, 0.20)
2020Q4	2.50 (2.10, 2.94)	0.11 (0.06, 0.16)	0.13 (0.08, 0.17)
2021Q1	2.67 (2.26, 3.12)	0.07 (0.03, 0.12)	0.11 (0.06, 0.15)
2021Q2	2.50 (2.10, 2.94)	0.09 (0.05, 0.14)	0.12 (0.08, 0.17)
2021Q3	0.48 (0.25, 0.73)	0.13 (0.08, 0.18)	0.16 (0.11, 0.20)
2021Q4	0.27 (0.06, 0.50)	0.11 (0.07, 0.16)	0.14 (0.10, 0.19)

CI, confidence interval; LTC, long-term care; NA, nursing assistant; RN, registered nurse.

^a^Values back-transformed from natural log scale, representing absolute changes in days per 100 inpatient-days; adjusted for facility factors (region, bed capacity, bed-to-RN ratio, and bed-to-NA ratio) and patient factors (age, gender, number of chronic diseases, presence of psychiatric diseases, and presence of neurological diseases).

All comparisons were statistically significant (*p* < 0.0001; Bonferroni-corrected *α* = 0.0013).

##### Antipsychotics

Quetiapine showed the greatest increase across all quarters, reaching an additional 1.30 days per 100 inpatient-days (*p* < 0.0001), followed by risperidone, with a maximum increase of approximately 0.03 days (*p* < 0.0001). Haloperidol demonstrated an early rise (< 0.01 days), followed by a gradual decline. Olanzapine and aripiprazole showed statistically significant but minimal increases across most quarters, whereas clozapine showed no significant change over the entire study period.

##### Anxiolytics

Lorazepam and alprazolam significantly increased across all quarters, with maximum rises of approximately 0.04 and 0.02 additional days, respectively (all *p* < 0.0001). Diazepam showed significant but smaller increases only during the first three quarters of 2020 (all *p* < 0.0001).

##### Antidepressants

Trazodone and escitalopram showed significant increases, peaking at approximately 0.04 and 0.02 additional days, respectively (all *p* < 0.0001). Sertraline and mirtazapine also increased slightly but consistently across most quarters.

#### Prescription doses (DDDs)

After adjusting for facility and patient-level factors, prescription DDDs increased for all psychotropic classes in every quarter compared with the pre-pandemic baseline (Table 3; Figure 2B; all *p* < 0.0001). Consistent with the findings for prescription days, antipsychotics showed the largest increase, peaking in early 2021 and remaining above baseline through 2021Q4, although diminishing in later quarters. Anxiolytic DDDs increased throughout the pandemic, especially during the early quarters of 2020, while antidepressant DDDs exhibited a steady rise over the study period. Drug-specific results are provided in Supplementary Table S3 and Supplementary Figures S1B–S3B.

**Table 3:** Adjusted linear mixed model estimates of changes in prescription doses by psychotropic class compared with the 2019Q4 baseline (per 100 inpatient-days).

Prescribing quarter	Antipsychotics	Anxiolytics	Antidepressants
	Mean difference (95% CI) ^a^	Mean difference (95% CI)^a^	Mean difference (95% CI) ^a^
2020Q1	0.36 (0.28, 0.45)	0.10 (0.07, 0.14)	0.09 (0.06, 0.12)
2020Q2	0.43 (0.35, 0.53)	0.10 (0.07, 0.13)	0.09 (0.07, 0.12)
2020Q3	0.48 (0.39, 0.58)	0.10 (0.07, 0.13)	0.10 (0.07, 0.12)
2020Q4	0.53 (0.44, 0.64)	0.07 (0.04, 0.10)	0.08 (0.06, 0.11)
2021Q1	0.57 (0.48, 0.68)	0.05 (0.02, 0.08)	0.08 (0.05, 0.10)
2021Q2	0.54 (0.44, 0.64)	0.06 (0.03, 0.09)	0.08 (0.05, 0.11)
2021Q3	0.13 (0.07, 0.21)	0.08 (0.05, 0.11)	0.10 (0.07, 0.13)
2021Q4	0.09 (0.03, 0.16)	0.07 (0.05, 0.11)	0.09 (0.07, 0.12)

CI, confidence interval; LTC, long-term care; NA, nursing assistant; RN, registered nurse.

^a^Values back-transformed from natural log scale, representing absolute changes in defined daily doses (DDDs) per 100 inpatient-days; adjusted for facility factors (region, bed capacity, bed-to-RN ratio, and bed-to-NA ratio) and patient factors (age, gender, number of chronic diseases, presence of psychiatric diseases, and presence of neurological diseases).

All comparisons were statistically significant (*p* < 0.0001; Bonferroni-corrected *α* = 0.0013).

##### Antipsychotics

Quetiapine showed the greatest increase across all quarters, peaking at 0.30 additional DDDs per 100 inpatient-days (*p* < 0.0001), followed by risperidone, which peaked at 0.02 additional DDDs (*p* < 0.0001). Haloperidol showed an early rise (< 0.01 DDDs) but gradually declined thereafter. Olanzapine and aripiprazole showed consistently significant but minimal increases throughout most quarters, whereas clozapine exhibited no significant change across all periods.

##### Anxiolytics

Lorazepam and alprazolam demonstrated significant increases across all quarters, peaking at 0.03 and 0.02 additional DDDs, respectively (all *p* < 0.0001). Diazepam showed significant but smaller increases only during the first three quarters of 2020 and in 2021Q3 (all *p* < 0.0001).

##### Antidepressants

Trazodone and escitalopram exhibited significant increases across all quarters, with maximum rises of 0.02 additional DDDs (all *p* < 0.0001). Sertraline and mirtazapine showed modest but statistically significant increases throughout most quarters.

## Discussion

In this nationally representative study, psychotropic prescription days and doses in Korean LTC hospitals increased during the pandemic and remained above the pre-pandemic baseline through 2021, although increases attenuated in late 2021, particularly for antipsychotics. Beyond reflecting short-term pandemic-related constraints on care delivery, persistent increases in psychotropic use after the early pandemic period suggest that the pandemic may have magnified pre-existing reliance on pharmacologic symptom management and underscores vulnerabilities in sustaining person-centered, non-pharmacological dementia care in LTC hospitals under crisis conditions.

LTC systems in North America and Europe are predominantly residential and socially oriented and have implemented policies that support antipsychotic stewardship and non-pharmacological dementia care, such as the U. S CMS National Partnership, Canada’s nationwide monitoring system on Potentially Inappropriate Use of Antipsychotics in Long-Term Care, and the U. K. National Dementia Strategy (5, 6, 34). In contrast, Korean LTC hospitals operate under a medicalized, insurance-integrated model (9), and national efforts to promote non-pharmacological interventions and minimize inappropriate psychotropic use appear less extensive. During the pandemic, LTC hospitals may have experienced heightened workloads and staffing strain, along with reductions in social engagement, structured activities, and family involvement (16, 18), leading to fewer opportunities for labor-intensive psychosocial care. These constraints could have increased pressure to use psychotropics as a readily available option for behavioral symptoms, potentially contributing to sustained increases in prescribing beyond the early pandemic period.

Class-specific patterns provide further insight. Antipsychotic use exhibited the most pronounced increase, consistent with prior studies (22, 35). Increases were particularly notable for quetiapine and risperidone, which are commonly used for BPSD and often perceived to have a lower risk of extrapyramidal side effects than some antipsychotics (36–38). A short-term increase in haloperidol use early in the pandemic (2020Q1) may reflect acute management of severe agitation in a constrained care environment, despite its higher risk of adverse effects in older adults (3). Clozapine showed no significant changes across periods, possibly reflecting clinicians’ caution regarding its association with agranulocytosis and metabolic complications (39).

A consistent increase in anxiolytic use was observed throughout the pandemic, with the largest rise in 2020Q1 and Q2. This pattern suggests increased use of anxiolytics, including benzodiazepines, for symptoms such as anxiety, agitation, and sleep disturbance, whereas some earlier studies reported stable or decreased anxiolytic/benzodiazepine use during the pandemic (21, 22). Alprazolam and lorazepam increased more than diazepam, potentially aligning with guidance to avoid long-acting benzodiazepines in older adults (2). Alprazolam and lorazepam, as short-acting benzodiazepines, are used for disruptive behaviors and sleep disturbances; however, long-term use can lead to dependence and withdrawal symptoms (40, 41).

Our findings indicated increased antidepressant use, consistent with prior studies reporting similar increases during the pandemic period in LTC settings (20–22). In our study, increases were greater for trazodone and escitalopram than for sertraline and mirtazapine. Trazodone may have been used to address sleep disturbance and associated nighttime agitation as an alternative to antipsychotics (42–44). Escitalopram may reflect treatment for pandemic-related anxiety and depressive symptoms (45). Together, these patterns are consistent with potential shifts in use across psychotropic classes and suggest that stewardship efforts may need to extend beyond antipsychotics.

### Strengths and limitations

A major strength of this study is the use of nationwide Korean National Health Insurance Service (NHIS) data to examine longitudinal patterns of psychotropic use in LTC hospitals before and during the COVID-19 pandemic. By capturing both prescription days and dose-based measures (DDDs) at the national level within a single-payer system, our findings provide insight into psychotropic stewardship needs in Korea’s medicalized LTC hospital model.

However, some limitations should be considered. The NHIS data lacked information on the frequency and severity of BPSD, non-pharmacological care delivery, or the clinical appropriateness of psychotropic prescribing in LTC hospital residents. Psychotropic prescribing may be clinically indicated when symptoms become unresponsive to non-pharmacological approaches and/or pose a substantial risk of harm to self and/or others (2). Also, we lacked staff-related information that may influence prescribing, including staff knowledge of BPSD care and appropriate psychotropic use, burnout, and distress levels (46, 47). Although we adjusted for facility-level bed-to-RN and bed-to-NA ratios, these structural staffing measures may not fully capture actual staffing availability, shift-level deployment, workload, or care processes relevant to non-pharmacological responses to BPSD. In addition, while physicians are routinely present and responsible for psychotropic prescribing in Korean LTC hospitals, physician staffing was not included as a covariate in the present analyses. The inclusion of random intercepts for LTC hospitals accounted for clustering within facilities and partially captured unmeasured between-facility heterogeneity, but it could not fully address unmeasured facility-level factors. Future studies incorporating workforce capacity, staff competencies, and care-process measures are needed to better understand prescribing trends and identify targets for improvement.

### Implications

Korea’s HIRA has published guidance on antipsychotic use for older adults with dementia (48). However, stewardship efforts may also need to address other commonly used psychotropics that may substitute for antipsychotics in practice. For example, benzodiazepines were reported as the most frequently prescribed potentially inappropriate medications among patients with dementia in Korea (49). Given ongoing concerns about adverse effects, long-term use of benzodiazepines and some antidepressants remains controversial, underscoring the need for further research to inform evidence-based prescribing and deprescribing practices.

Strengthening the resilience of dementia care in LTC hospitals may require integrating crisis-contingent strategies into practice to protect psychosocial, non-pharmacological care during public health emergencies. Priorities include adequate nurse staffing, digital or remote non-pharmacological interventions (e.g., tele-activity programs, virtual family contact, online cognitive engagement), and routine psychotropic monitoring systems that track initiation, duration, dose escalation, and deprescribing. In addition, policies to monitor inappropriate psychotropic use, such as quality oversight approaches in U.S. nursing homes (e.g., deficiency citations for unnecessary psychotropic use), could be considered and adopted for Korean LTC hospital settings to enhance safety and quality dementia care during future crises (50).

## Conclusion

This national longitudinal study found that psychotropic use among older adults with dementia in Korean LTC hospitals increased during the pandemic period and remained above pre-pandemic baseline levels through 2021, although these increases diminished substantially in the later quarters of 2021, particularly for antipsychotics. Persistent higher use suggests increased reliance on pharmacologic symptom management when routine psychosocial dementia care was constrained. The findings underscore vulnerabilities in maintaining continuity of non-pharmacological care during public health emergencies and the need for crisis-resilient, person-centered dementia care in LTC hospitals. Routine psychotropic stewardship and monitoring should track not only antipsychotics but also commonly used alternatives, including benzodiazepines and antidepressants, across initiation, duration, dose escalation, and deprescribing. Future research should examine how these prescribing patterns relate to patient outcomes and whether elevated psychotropic use persists beyond the pandemic period.

## Data Availability

The datasets presented in this article are not readily available because the data analyzed in this study are derived from the Korean National Health Insurance Service (NHIS) claims database and were provided as customized, de-identified data under an approved data request. Due to legal and data protection restrictions, the datasets are not publicly available and cannot be shared by the authors. Researchers who meet the criteria for access may request the data directly from NHIS through its data access procedures. Requests to access the datasets should be directed to NHIS-Big Data Platform, https://nhiss.nhis.or.kr/en/z/a/001/lpza001m01en.do.
