# Supplementary material for: Trends in psychotropic use among older adults with dementia in Korean long-term care hospitals across the COVID-19 pandemic: a national longitudinal study
Source: Front Public Health. 2026 Jun 15;14:1776066. doi: 10.3389/fpubh.2026.1776066 (PMC13310674; doi:10.3389/fpubh.2026.1776066)
Supplement: Supplementary file 1 [file table_1.docx]

**Supplementary materials**

**Table S1.** Diagnostic codes from the seventh and eighth editions of the Korean Standard Classification of Diseases (KCD-7 and KCD-8) for defining chronic diseases, psychiatric diseases, and neurological diseases.

**Table S2.** Adjusted linear mixed model estimates of changes in prescription days for psychotropic drugs compared with the 2019Q4 baseline (per 100 inpatient-days).

**Table S3.** Adjusted linear mixed model estimates of changes in prescription doses for psychotropic drugs compared with the 2019Q4 baseline (per 100 inpatient-days).

**Figure S1.** Adjusted changes in prescription days and doses by antipsychotic drug compared with the 2019Q4 baseline (per 100 inpatient-days).

**Figure S2.** Adjusted changes in prescription days and doses by anxiolytic drug compared with the 2019Q4 baseline (per 100 inpatient-days).

**Figure S3.** Adjusted changes in prescription days and doses by antidepressant drug compared with the 2019Q4 baseline (per 100 inpatient-days).

**Table S1.** Diagnostic codes from the seventh and eighth editions of the Korean Standard Classification of Diseases (KCD-7 and KCD-8) for defining chronic diseases, psychiatric diseases, and neurological diseases.

| Variable | KCD codes |
| --- | --- |
| **Number of chronic diseases (range: 1–13)** |  |
| 1. Hypertensive diseases | I10–I15 |
| 1. Diabetes mellitus | E10–E14 |
| 1. Mental and behavioral disorders | F00–F99 ^†^, G40–G41 |
| 1. Respiratory tuberculosis | A15, A16, A19 |
| 1. Heart diseases | I05–I09, I20–I27, I30–I52 |
| 1. Cerebrovascular diseases | I60–I69 |
| 1. Neurologic diseases | G00–G37 ^‡^, G43–G83 |
| 1. Neoplasms | C00–C97, D00–D09, D32–D33, D37–D48 |
| 1. Disorders of thyroid gland | E00–E07 |
| 1. Liver diseases | B18, B19, K70–K77 |
| 1. Chronic kidney disease | N18 |
| 1. Arthropathies | M00–M09, M11–M19, M45 |
| 1. COVID-19 infection | B342, U071, U181 |
| **Presence of psychiatric diseases (yes/no)** | F00–F99 ^†^, G40–G41, G470, R258, G256, G500, G501, T652, Z726 |
| **Presence of neurological diseases (yes/no)** | G00–G99 ^‡^ |

^†^ Excluding diagnostic codes defined as dementia in this study: F00~F03, F051.

^‡^ Excluding diagnostic codes defined as dementia in this study: G30, G3100–G3104, G3182.

**Table S2.** Adjusted linear mixed model estimates of changes in prescription days for psychotropic drugs compared with the 2019Q4 baseline (per 100 inpatient-days).

1. Antipsychotics

| Prescribing quarter | Aripiprazole | | Clozapine | | Haloperidol | |
| --- | --- | --- | --- | --- | --- | --- |
|  | Mean difference (95% CI) ^a^ | *p* value ^b^ | Mean difference (95% CI) ^a^ | *p* value ^b^ | Mean difference (95% CI) ^a^ | *p* value ^b^ |
| 2020Q1 | 0.000 (-0.002, 0.003) | .0062 | 0.000 (-0.001, 0.001) | .1594 | **0.009 (0.005, 0.013)** | **< .0001** |
| 2020Q2 | 0.001 (-0.002, 0.003) | .0001 | 0.000 (-0.001, 0.001) | .0987 | **0.006 (0.003, 0.010)** | **< .0001** |
| 2020Q3 | **0.001 (-0.001, 0.003)** | **< .0001** | 0.000 (0.001, 0.001) | .0660 | **0.006 (0.002, 0.010)** | **< .0001** |
| 2020Q4 | **0.001 (-0.001, 0.003)** | **< .0001** | 0.000 (0.001, 0.001) | .0174 | **0.003 (-0.001, 0.007)** | **< .0001** |
| 2021Q1 | **0.001 (-0.001, 0.003)** | **< .0001** | 0.000 (0.001, 0.001) | .0169 | **0.002 (-0.001, 0.006)** | **< .0001** |
| 2021Q2 | **0.001 (-0.001, 0.003)** | **< .0001** | 0.000 (0.001, 0.001) | .0090 | 0.002 (-0.002, 0.005) | .0016 |
| 2021Q3 | **0.001 (-0.001, 0.004)** | **< .0001** | 0.000 (-0.001, 0.001) | .1504 | **-0.006 (-0.010, -0.002)** | **< .0001** |
| 2021Q4 | **0.001 (-0.001, 0.004)** | **< .0001** | 0.000 (-0.001, 0.001) | .1507 | **-0.007 (-0.010, -0.003)** | **< .0001** |
| Prescribing quarter | Olanzapine | | Quetiapine | | Risperidone | |
|  | Mean difference (95% CI) ^a^ | *p* value ^b^ | Mean difference (95% CI) ^a^ | *p* value ^b^ | Mean difference (95% CI) ^a^ | *p* value ^b^ |
| 2020Q1 | **0.001 (-0.003, 0.005)** | **< .0001** | **0.635 (0.483, 0.802)** | **< .0001** | **0.032 (0.019, 0.044)** | **< .0001** |
| 2020Q2 | **0.001 (-0.003, 0.005)** | **< .0001** | **0.850 (0.680, 1.035)** | **< .0001** | **0.033 (0.021, 0.046)** | **< .0001** |
| 2020Q3 | **0.001 (-0.003, 0.005)** | **< .0001** | **1.004 (0.821, 1.203)** | **< .0001** | **0.032 (0.020, 0.045)** | **< .0001** |
| 2020Q4 | **0.001 (-0.003, 0.005)** | **< .0001** | **1.205 (1.006, 1.422)** | **< .0001** | **0.032 (0.020, 0.045)** | **< .0001** |
| 2021Q1 | **0.001 (-0.003, 0.006)** | **< .0001** | **1.300 (1.093, 1.525)** | **< .0001** | **0.033 (0.020, 0.045)** | **< .0001** |
| 2021Q2 | **0.002 (-0.003, 0.006)** | **< .0001** | **1.250 (1.048, 1.471)** | **< .0001** | **0.032 (0.020, 0.045)** | **< .0001** |
| 2021Q3 | **0.001 (-0.003, 0.006)** | **< .0001** | **0.289 (0.164, 0.424)** | **< .0001** | **0.007 (-0.004, 0.018)** | **< .0001** |
| 2021Q4 | **0.001 (-0.003, 0.006)** | **< .0001** | **0.199 (0.082, 0.326)** | **< .0001** | 0.002 (-0.009, 0.013) | .1933 |

*Note.* CI = confidence interval; LTC = long-term care; NA = nursing assistant; RN = registered nurse.

^a^ Values back-transformed from natural log scale, representing absolute changes in prescription days per 100 inpatient-days; adjusted for facility factors (region, bed capacity, bed-to-RN ratio, and bed-to-NA ratio) and patient factors (age, gender, number of chronic diseases, presence of psychiatric diseases, and presence of neurological diseases).

^b^ Statistically significant at Bonferroni-corrected *α* = 0.0013 (indicated in bold).

**Table S2.** Adjusted linear mixed model estimates of changes in prescription days for psychotropic drugs compared with the 2019Q4 baseline (per 100 inpatient-days) (continued).

1. Anxiolytics

| Prescribing quarter | Alprazolam | | Diazepam | | Lorazepam | |
| --- | --- | --- | --- | --- | --- | --- |
|  | Mean difference (95% CI) ^a^ | *p* value ^b^ | Mean difference (95% CI) ^a^ | *p* value ^b^ | Mean difference (95% CI) ^a^ | *p* value ^b^ |
| 2020Q1 | **0.022 (0.013, 0.031)** | **< .0001** | **0.006 (0.003, 0.009)** | **< .0001** | **0.029 (0.012, 0.047)** | **< .0001** |
| 2020Q2 | **0.024 (0.051, 0.033)** | **< .0001** | **0.005 (0.002, 0.008)** | **< .0001** | **0.028 (0.012, 0.046)** | **< .0001** |
| 2020Q3 | **0.021 (0.012, 0.030)** | **< .0001** | **0.004 (0.001, 0.007)** | **< .0001** | **0.028 (0.011, 0.046)** | **< .0001** |
| 2020Q4 | **0.014 (0.006, 0.023)** | **< .0001** | 0.001 (-0.002, 0.004) | .0118 | **0.020 (0.004, 0.038)** | **< .0001** |
| 2021Q1 | **0.010 (0.002, 0.018)** | **< .0001** | 0.000 (-0.003, 0.003) | .6420 | **0.019 (0.003, 0.036)** | **< .0001** |
| 2021Q2 | **0.013 (0.005, 0.022)** | **< .0001** | 0.000 (-0.003, 0.003) | .3977 | **0.026 (0.009, 0.044)** | **< .0001** |
| 2021Q3 | **0.016 (0.007, 0.025)** | **< .0001** | 0.001 (-0.002, 0.004) | .0086 | **0.035 (0.018, 0.053)** | **< .0001** |
| 2021Q4 | **0.014 (0.005, 0.023)** | **< .0001** | 0.000 (-0.003, 0.003) | .9357 | **0.033 (0.016, 0.052)** | **< .0001** |

*Note.* CI = confidence interval; LTC = long-term care; NA = nursing assistant; RN = registered nurse.

^a^ Values back-transformed from natural log scale, representing absolute changes in prescription days per 100 inpatient-days; adjusted for facility factors (region, bed capacity, bed-to-RN ratio, and bed-to-NA ratio) and patient factors (age, gender, number of chronic diseases, presence of psychiatric diseases, and presence of neurological diseases).

^b^ Statistically significant at Bonferroni-corrected *α* = 0.0013 (indicated in bold).

**Table S2.** Adjusted linear mixed model estimates of changes in prescription days for psychotropic drugs compared with the 2019Q4 baseline (per 100 inpatient-days) (continued).

1. Antidepressants

| Prescribing quarter | Escitalopram | | Mirtazapine | | Sertraline | |
| --- | --- | --- | --- | --- | --- | --- |
|  | Mean difference (95% CI) ^a^ | *p* value ^b^ | Mean difference (95% CI) ^a^ | *p* value ^b^ | Mean difference (95% CI) ^a^ | *p* value ^b^ |
| 2020Q1 | **0.014 (0.006, 0.022)** | **< .0001** | 0.001 (-0.001, 0.002) | .0114 | **0.001 (-0.001, 0.002)** | **< .0001** |
| 2020Q2 | **0.016 (0.008, 0.024)** | **< .0001** | **0.001 (-0.001, 0.002)** | **.0009** | **0.001 (-0.001, 0.002)** | **.0001** |
| 2020Q3 | **0.018 (0.010, 0.026)** | **< .0001** | **0.001 (-0.001, 0.002)** | **.0006** | **0.001 (0.000, 0.002)** | **< .0001** |
| 2020Q4 | **0.018 (0.010, 0.026)** | **< .0001** | 0.001 (-0.001, 0.002) | .0024 | **0.001 (0.000, 0.002)** | **< .0001** |
| 2021Q1 | **0.018 (0.010, 0.026)** | **< .0001** | 0.001 (-0.001, 0.002) | .0014 | **0.001 (0.000, 0.003)** | **< .0001** |
| 2021Q2 | **0.019 (0.011, 0.027)** | **< .0001** | **0.001 (-0.001, 0.003)** | **.0002** | **0.001 (0.000, 0.003)** | **< .0001** |
| 2021Q3 | **0.022 (0.014, 0.030)** | **< .0001** | **0.001 (0.001, 0.003)** | **< .0001** | **0.001 (0.000, 0.003)** | **< .0001** |
| 2021Q4 | **0.022 (0.014, 0.030)** | **< .0001** | **0.001 (0.001, 0.003)** | **< .0001** | **0.001 (0.000, 0.002)** | **< .0001** |
| Prescribing quarter | Trazodone | |  | |  | |
|  | Mean difference (95% CI) ^a^ | *p* value ^b^ |  |  |  |  |
| 2020Q1 | **0.026 (0.013, 0.039)** | **< .0001** |  |  |  |  |
| 2020Q2 | **0.026 (0.014, 0.040)** | **< .0001** |  |  |  |  |
| 2020Q3 | **0.023 (0.010, 0.036)** | **< .0001** |  |  |  |  |
| 2020Q4 | **0.018 (0.006, 0.031)** | **< .0001** |  |  |  |  |
| 2021Q1 | **0.015 (0.003, 0.027)** | **< .0001** |  |  |  |  |
| 2021Q2 | **0.022 (0.010, 0.035)** | **< .0001** |  |  |  |  |
| 2021Q3 | **0.035 (0.022, 0.048)** | **< .0001** |  |  |  |  |
| 2021Q4 | **0.033 (0.020, 0.047)** | **< .0001** |  |  |  |  |

*Note.* CI = confidence interval; LTC = long-term care; NA = nursing assistant; RN = registered nurse.

^a^ Values back-transformed from natural log scale, representing absolute changes in prescription days per 100 inpatient-days; adjusted for facility factors (region, bed capacity, bed-to-RN ratio, and bed-to-NA ratio) and patient factors (age, gender, number of chronic diseases, presence of psychiatric diseases, and presence of neurological diseases).

^b^ Statistically significant at Bonferroni-corrected *α* = 0.0013 (indicated in bold).

**Table S3.** Adjusted linear mixed model estimates of changes in prescription doses for psychotropic drugs compared with the 2019Q4 baseline (per 100 inpatient-days).

1. Antipsychotics

| Prescribing quarter | Aripiprazole | | Clozapine | | Haloperidol | |
| --- | --- | --- | --- | --- | --- | --- |
|  | Mean difference (95% CI) ^a^ | *p* value ^b^ | Mean difference (95% CI) ^a^ | *p* value ^b^ | Mean difference (95% CI) ^a^ | *p* value ^b^ |
| 2020Q1 | 0.000 (-0.002, 0.002) | .0040 | 0.000 (0.000, 0.001) | .1432 | **0.007 (0.004, 0.010)** | **< .0001** |
| 2020Q2 | **0.000 (-0.001, 0.002)** | **.0001** | 0.000 (0.000, 0.001) | .1047 | **0.005 (0.002, 0.008)** | **< .0001** |
| 2020Q3 | **0.001 (-0.001, 0.003)** | **< .0001** | 0.000 (0.000, 0.001) | .0663 | **0.004 (0.001, 0.007)** | **< .0001** |
| 2020Q4 | **0.001 (-0.001, 0.003)** | **< .0001** | 0.000 (0.000, 0.001) | .0327 | **0.002 (-0.001, 0.005)** | **< .0001** |
| 2021Q1 | **0.001 (-0.001, 0.003)** | **< .0001** | 0.000 (0.000, 0.001) | .0261 | **0.002 (-0.001, 0.005)** | **< .0001** |
| 2021Q2 | **0.001 (-0.001, 0.003)** | **< .0001** | 0.000 (0.000, 0.001) | .0183 | **0.001 (-0.002, 0.004)** | **.0009** |
| 2021Q3 | **0.001 (-0.001, 0.003)** | **< .0001** | 0.000 (0.000, 0.001) | .1672 | **-0.004 (-0.007, -0.002)** | **< .0001** |
| 2021Q4 | **0.001 (-0.001, 0.003)** | **< .0001** | 0.000 (0.000, 0.001) | .1796 | **-0.005 (-0.008, -0.002)** | **< .0001** |
| Prescribing quarter | Olanzapine | | Quetiapine | | Risperidone | |
|  | Mean difference (95% CI) ^a^ | *p* value ^b^ | Mean difference (95% CI) ^a^ | *p* value ^b^ | Mean difference (95% CI) ^a^ | *p* value ^b^ |
| 2020Q1 | **0.001 (-0.003, 0.005)** | **< .0001** | **0.167 (0.132, 0.205)** | **< .0001** | **0.020 (0.012, 0.027)** | **< .0001** |
| 2020Q2 | **0.001 (-0.003, 0.005)** | **< .0001** | **0.214 (0.176, 0.255)** | **< .0001** | **0.020 (0.013, 0.028)** | **< .0001** |
| 2020Q3 | **0.001 (-0.003, 0.005)** | **< .0001** | **0.241 (0.202, 0.284)** | **< .0001** | **0.019 (0.012, 0.027)** | **< .0001** |
| 2020Q4 | **0.001 (-0.003, 0.005)** | **< .0001** | **0.274 (0.232, 0.318)** | **< .0001** | **0.019 (0.011, 0.027)** | **< .0001** |
| 2021Q1 | **0.001 (-0.003, 0.005)** | **< .0001** | **0.296 (0.253, 0.341)** | **< .0001** | **0.020 (0.012, 0.028)** | **< .0001** |
| 2021Q2 | **0.001 (-0.002, 0.005)** | **< .0001** | **0.286 (0.244, 0.331)** | **< .0001** | **0.019 (0.012, 0.027)** | **< .0001** |
| 2021Q3 | **0.001 (-0.003, 0.005)** | **< .0001** | **0.087 (0.055, 0.120)** | **< .0001** | **0.005 (-0.002, 0.012)** | **< .0001** |
| 2021Q4 | **0.001 (-0.002, 0.005)** | **< .0001** | **0.071 (0.040, 0.103)** | **< .0001** | 0.002 (-0.005, 0.009) | .0114 |

*Note.* CI = confidence interval; LTC = long-term care; NA = nursing assistant; RN = registered nurse.

^a^ Values back-transformed from natural log scale, representing absolute changes in prescription doses per 100 inpatient-days; adjusted for facility factors (region, bed capacity, bed-to-RN ratio, and bed-to-NA ratio) and patient factors (age, gender, number of chronic diseases, presence of psychiatric diseases, and presence of neurological diseases).

^b^ Statistically significant at Bonferroni-corrected *α* = 0.0013 (indicated in bold).**Table S3.** Adjusted linear mixed model estimates of changes in prescription doses for psychotropic drugs compared with the 2019Q4 baseline (per 100 inpatient-days) (continued).

1. Anxiolytics

| Prescribing quarter | Alprazolam | | Diazepam | | Lorazepam | |
| --- | --- | --- | --- | --- | --- | --- |
|  | Mean difference (95% CI) ^a^ | *p* value ^b^ | Mean difference (95% CI) ^a^ | *p* value ^b^ | Mean difference (95% CI) ^a^ | *p* value ^b^ |
| 2020Q1 | **0.016 (0.010, 0.023)** | **< .0001** | **0.005 (0.003, 0.007)** | **< .0001** | **0.022 (0.009, 0.036)** | **< .0001** |
| 2020Q2 | **0.018 (0.012, 0.024)** | **< .0001** | **0.004 (0.002, 0.006)** | **< .0001** | **0.022 (0.009, 0.036)** | **< .0001** |
| 2020Q3 | **0.016 (0.010, 0.022)** | **< .0001** | **0.003 (0.001, 0.006)** | **< .0001** | **0.021 (0.008, 0.035)** | **< .0001** |
| 2020Q4 | **0.011 (0.005, 0.017)** | **< .0001** | 0.001 (-0.001, 0.003) | .0037 | **0.016 (0.003, 0.029)** | **< .0001** |
| 2021Q1 | **0.008 (0.002, 0.014)** | **< .0001** | 0.000 (-0.002, 0.003) | .1734 | **0.015 (0.002, 0.029)** | **< .0001** |
| 2021Q2 | **0.010 (0.004, 0.016)** | **< .0001** | 0.001 (-0.002, 0.003) | .1180 | **0.020 (0.007, 0.034)** | **< .0001** |
| 2021Q3 | **0.012 (0.006, 0.019)** | **< .0001** | **0.001 (-0.001, 0.004)** | **.0002** | **0.027 (0.014, 0.041)** | **< .0001** |
| 2021Q4 | **0.011 (0.005, 0.017)** | **< .0001** | 0.001 (-0.002, 0.003) | .1433 | **0.026 (0.013, 0.040)** | **< .0001** |

*Note.* CI = confidence interval; LTC = long-term care; NA = nursing assistant; RN = registered nurse.

^a^ Values back-transformed from natural log scale, representing absolute changes in doses per 100 inpatient-days; adjusted for facility factors (region, bed capacity, bed-to-RN ratio, and bed-to-NA ratio) and patient factors (age, gender, number of chronic diseases, presence of psychiatric diseases, and presence of neurological diseases).

^b^ Statistically significant at Bonferroni-corrected *α* = 0.0013 (indicated in bold).

**Table S3.** Adjusted linear mixed model estimates of changes in prescription doses for psychotropic drugs compared with the 2019Q4 baseline (per 100 inpatient-days) (continued).

1. Antidepressants

| Prescribing quarter | Escitalopram | | Mirtazapine | | Sertraline | |
| --- | --- | --- | --- | --- | --- | --- |
|  | Mean difference (95% CI) ^a^ | *p* value ^b^ | Mean difference (95% CI) ^a^ | *p* value ^b^ | Mean difference (95% CI) ^a^ | *p* value ^b^ |
| 2020Q1 | **0.013 (0.006, 0.021)** | **< .0001** | 0.000 (-0.001, 0.002) | .0049 | **0.001 (-0.001, 0.002)** | **.0001** |
| 2020Q2 | **0.015 (0.007, 0.023)** | **< .0001** | **0.001 (-0.001, 0.002)** | **.0004** | **0.001 (-0.001, 0.002)** | **.0001** |
| 2020Q3 | **0.017 (0.009, 0.025)** | **< .0001** | **0.001 (-0.001, 0.002)** | **.0003** | **0.001 (0.000, 0.002)** | **< .0001** |
| 2020Q4 | **0.017 (0.010, 0.025)** | **< .0001** | 0.001 (-0.001, 0.002) | .0015 | **0.001 (0.000, 0.002)** | **< .0001** |
| 2021Q1 | **0.017 (0.009, 0.025)** | **< .0001** | **0.001 (-0.001, 0.002)** | **.0008** | **0.001 (0.000, 0.003)** | **< .0001** |
| 2021Q2 | **0.018 (0.010, 0.026)** | **< .0001** | **0.001 (-0.001, 0.002)** | **.0001** | **0.001 (0.000, 0.003)** | **< .0001** |
| 2021Q3 | **0.021 (0.013, 0.029)** | **< .0001** | **0.001 (0.000, 0.003)** | **< .0001** | **0.001 (0.000, 0.003)** | **< .0001** |
| 2021Q4 | **0.021 (0.013, 0.029)** | **< .0001** | **0.001 (0.000, 0.003)** | **< .0001** | **0.001 (0.000, 0.002)** | **< .0001** |
| Prescribing quarter | Trazodone | |  | |  | |
|  | Mean difference (95% CI) ^a^ | *p* value ^b^ |  |  |  |  |
| 2020Q1 | **0.015 (0.009, 0.022)** | **< .0001** |  |  |  |  |
| 2020Q2 | **0.015 (0.009, 0.022)** | **< .0001** |  |  |  |  |
| 2020Q3 | **0.014 (0.007, 0.020)** | **< .0001** |  |  |  |  |
| 2020Q4 | **0.011 (0.005, 0.017)** | **< .0001** |  |  |  |  |
| 2021Q1 | **0.010 (0.004, 0.016)** | **< .0001** |  |  |  |  |
| 2021Q2 | **0.013 (0.007, 0.020)** | **< .0001** |  |  |  |  |
| 2021Q3 | **0.020 (0.013, 0.026)** | **< .0001** |  |  |  |  |
| 2021Q4 | **0.020 (0.013, 0.026)** | **< .0001** |  |  |  |  |

*Note.* CI = confidence interval; LTC = long-term care; NA = nursing assistant; RN = registered nurse.

^a^ Values back-transformed from natural log scale, representing absolute changes in prescription doses per 100 inpatient-days; adjusted for facility factors (region, bed capacity, bed-to-RN ratio, and bed-to-NA ratio) and patient factors (age, gender, number of chronic diseases, presence of psychiatric diseases, and presence of neurological diseases).

^b^ Statistically significant at Bonferroni-corrected *α* = 0.0013 (indicated in bold).


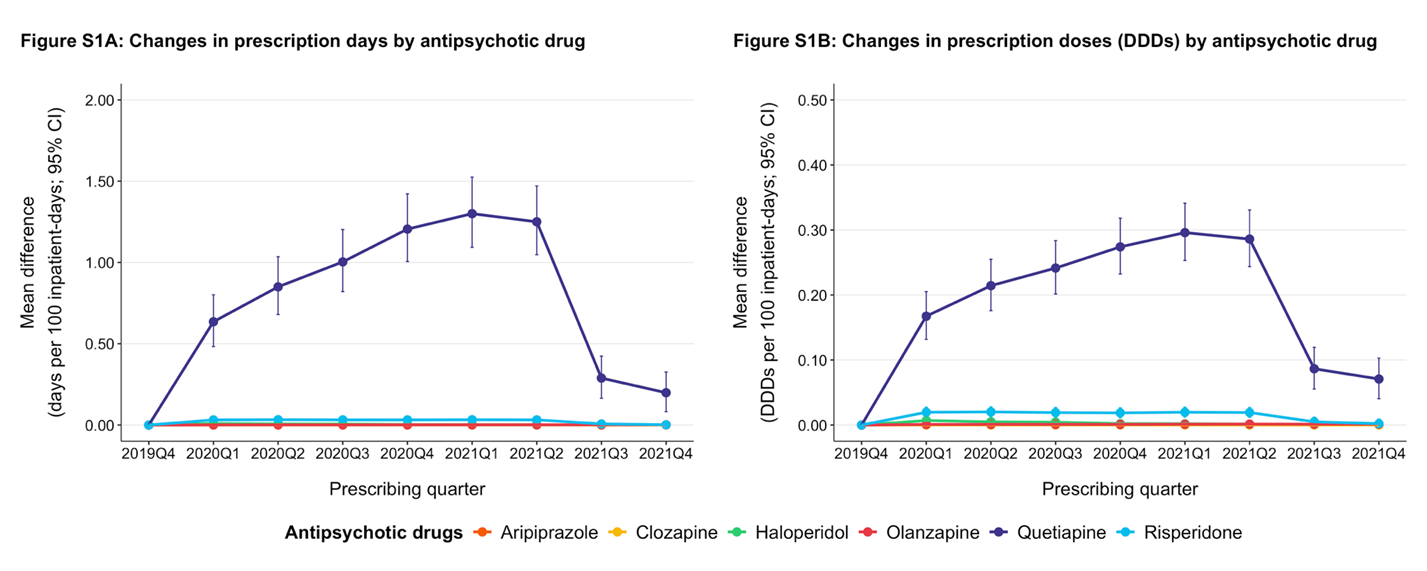


**Figure S1.** Adjusted changes in prescription days and doses by antipsychotic drug compared with the 2019Q4 baseline (per 100 inpatient-days).

*Note.* CI = confidence interval; DDDs = defined daily doses.


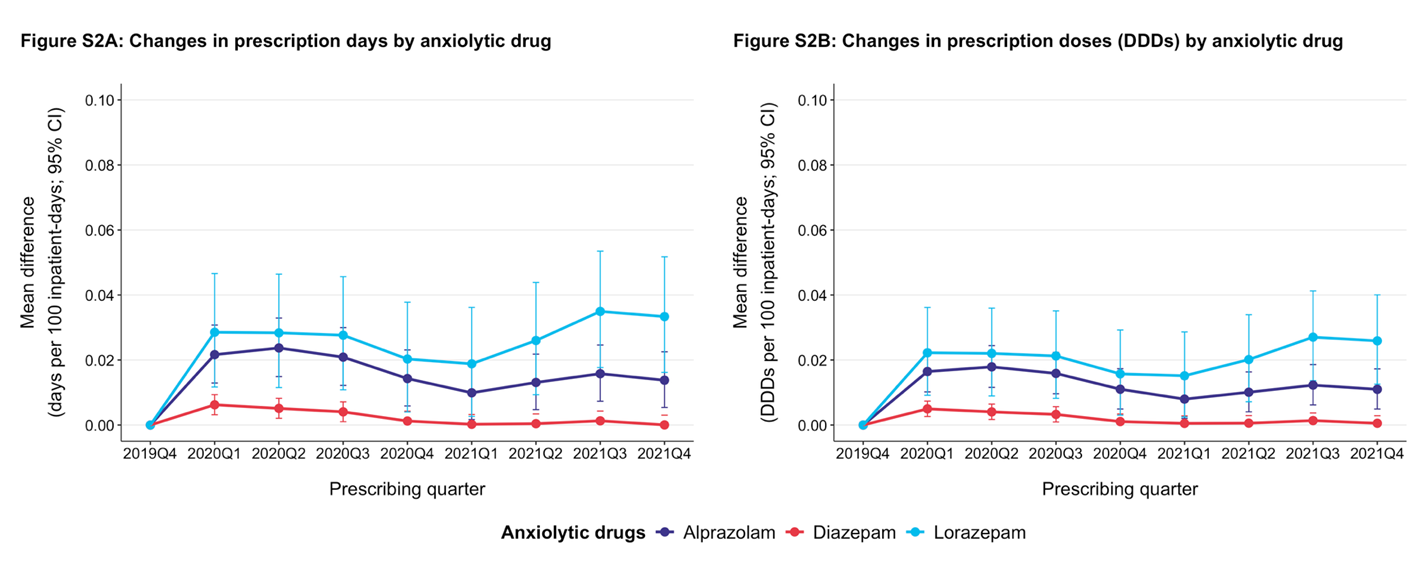


**Figure S2.** Adjusted changes in prescription days and doses by anxiolytic drug compared with the 2019Q4 baseline (per 100 inpatient-days).

*Note.* CI = confidence interval; DDDs = defined daily doses.


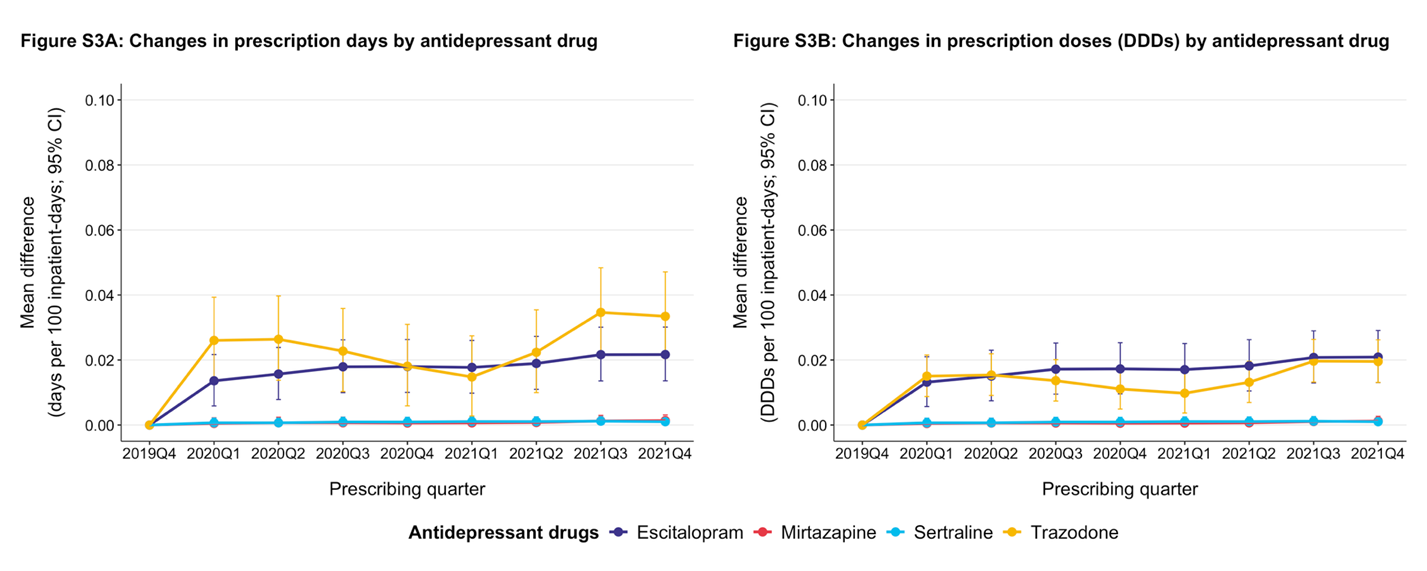


**Figure S3.** Adjusted changes in prescription days and doses by antidepressant drug compared with the 2019Q4 baseline (per 100 inpatient-days).

*Note.* CI = confidence interval; DDDs = defined daily doses.
